# An ecological economic comparison between integrated rice-fish farming and rice monocultures with low and high dikes in the Mekong Delta, Vietnam

**DOI:** 10.1007/s13280-023-01864-x

**Published:** 2023-05-15

**Authors:** Håkan Berg, Thai Huynh Phuong Lan, Nguyen Thanh Tam, Duong Huyen Trang, Pham Huynh Thanh Van, Huynh Ngoc Duc, Chau Thi Da

**Affiliations:** 1 Department of Physical Geography, Stockholm University, 106 91 Stockholm, Sweden; 2 Faculty of Agriculture and Natural Resources, An Giang University, 18 Ung Van Khiem, Long Xuyen City, An Giang 90000 Vietnam; 3 Faculty of Fishery, Nong Lam University, Block 6, Linh Trung Ward, Thu Duc District, HCM City, Vietnam; 4 Faculty of Applied Sciences, Ton Duc Thang University, 19 Nguyen Huu Tho street, District 7, Ho Chi Minh City, Vietnam

**Keywords:** Connectivity, Diversification, Food security, Integrated pest management, Pesticides, Sustainable agriculture

## Abstract

This study makes an ecological economic comparison between three different rice farming strategies in the Mekong Delta. Interviews were made with 30 farmers with two crops of rice and low dikes (2RLd), 30 farmers with three crops of rice and high dikes (3RHd) and 18 farmers with two crops of rice and one crop of fish (2RF). 2RF farmers had the highest annual net income and benefit cost ratio, because of low production costs and high yields of rice and fish. 2RLd farmers had the lowest annual net income. 3RHd had the highest annual rice yield, but also used the highest amount of rice seeds and agrochemicals, generating the lowest benefit cost ratio. Most farmers (70%) preferred two crops because of a higher production efficiency. High dikes and frequent use of pesticides and fertilizers were seen to decrease the water and rice quality, connectivity and biodiversity in farms with three crops. It is concluded that rice farming with two crops, and especially if integrated with fish and applying IPM, provides a sustainable alternative to rice farming with three crops and high dikes, because it makes use of the high connectivity within the rice-field ecosystem for an efficient production of healthy food through increased recycling of nutrients and  natural pest control mechanisms.

**Supplementary Information:**

The online version contains supplementary material available at 10.1007/s13280-023-01864-x.

## Introduction

The Mekong Delta covers only 12% of Vietnams area but supplies more than 50% of the country’s rice production (Tong 2017). Increased rice yields have been achieved through intensified farming methods, including increased use of agrochemicals (Berg et al. 2012) and constructions of high dikes to protect crops from floods (Tran et al. 2018; Hui et al. 2022).

This has increased income and food security but has also been followed by negative environmental impacts, such as pollution and biodiversity change (Tong 2017; Sebesvari et al. 2012; Nguyen et al. 2019; Spangenberg 2019). Tam et al. (2016) reported that farmers spraying organophosphates on rice fields resulted in both reduced growth and survival rates of fish and Dasgupta et al. (2007) found that over 35% of 190 rice farmers in the Mekong Delta, experienced acute pesticide poisoning.

Increased number of high dikes have decreased the aquatic connectivity within the Delta, followed by a decreased inflow of nutrient-rich water and sediments to agriculture areas and disrupted migration routes for fish (Tran et al. 2018, 2019; Nguyen et al. 2019; Dang et al. 2021). Increased reliance on agrochemicals and reduced exchange of water have also reduced the water quality in large parts of the Delta (Sebesvari et al. 2012). Expanded areas of double and triple rice production across the upper floodplains have halved the floodwater retention capacity compared to 2000, leading to greater flood risks downstream (Tran et al. 2019).

As a consequence, the benefits with intensive rice farming, which are dependent on high dikes and agrochemical inputs, are increasingly being questioned (Smajgl et al. 2015; Tran et al. 2018, 2019; Nguyen et al. 2018, 2019). The Mekong Delta is presently at a crossroad, where it either could enter a deteriorating state, or return to modified and more natural conditions (Tran et al. 2018). The direction it takes will depend largely on whether the intensification of rice production continues or if, instead, more sustainable farming strategies are adopted (Spangenberg 2019).

Such strategies include for example integrated rice-fish farming and integrated pest management (IPM), which have resulted in markedly improved rice farming systems (Xie et al. 2011; Berg et al. 2016; Zheng et al. 2016). These systems build on ecological principles and make use of the high connectivity and recycling of nutrient and matters within the rice field ecosystem, for an efficient and environmentally sound production of both rice and fish (Xie et al. 2011; Luo et al. 2014; Zheng et al. 2016). Low dikes allow fish to enter the rice field and a natural inflow of nutrient-rich water and sediments, which enrich the soil and decrease the farmers’ cost for synthetic fertilizers (Dung et al. 2018). Increased exchange of water and decreased use of agrochemicals increase the quality of water and create suitable living conditions for aquatic organisms and natural enemies to rice pests. Rice-fish farming with IPM strategies has been shown to give both higher rice and fish yields than non-IPM rice-fish farmers, and consumers are willing to pay a higher price for this rice because it is seen to be of higher quality (Khai and Jabe 2015; Berg and Tam 2018; Nguyen et al. 2018).

Thus, these farming strategies could provide sustainable options to intensive rice monocropping, with decreased impacts on the environment and the farmers health, which are in line with governmental efforts to introduce more sustainable farming systems in the Mekong Delta (Vietnamese government 2017, 2020). However, these policies are sometimes contradicted by believes that a continues high production of rice only can be achieved through increased use of agrochemicals (cf. Spangenberg 2019; Hutton et al 2021). The aim with the current study was, thus, to show that integrated systems, such as rice-fish farming, not only make ecological sense but also financial sense as they can increase the farmers’ income through decreased production costs and increased yields and price for both rice and fish (Berg and Tam 2018; Nguyen et al. 2018). Integrated rice-fish farming is part of Globally Important Agricultural Heritage Systems (GIAHS), which often are seen as more resilient and economically better than non-GIAHS options, and can thus provide examples of how to achieve sustainable diversification of food production systems in the Mekong Delta (Berweck et al. 2013; Garbach et al. 2014).

This study aims to assess if less intensive and more diverse rice farming strategies can provide sustainable alternatives to more intensive rice monocropping strategies for increased and diversified food production in the Dong Thap province in the Mekong Delta, Vietnam. Farms with two rice crops and low dikes were used as a baseline to compare the effects of conventional intensification through three rice crops and high dikes, with sustainable diversification through two rice crops and one crop of fish*.* By using these three different systems the aim was also to show how changed physical connectivity (impacted by high dikes) and ecological connectivity (impacted by pesticides) impact on ecosystem services, farmers profit and wellbeing. The underlying hypothesis of the study was that intensive rice farming systems manage agrochemicals and water suboptimal, which increases the production cost and decreases the yield of both rice and fish by disrupting the connectivity between and within the rice field ecosystems. Sustainable use of ecosystem services, such as natural pest control mechanisms (IPM) and fertilization by nutrient-rich flood water, however, can make rice-fish farming a financially competitive alternative to rice monocropping. This also helps farmers to diversify the production and decrease their use of agrochemicals which have positive impacts on the environment, farmers health and well-being.

## Materials and methods

### Study area

**Fig. 1:**
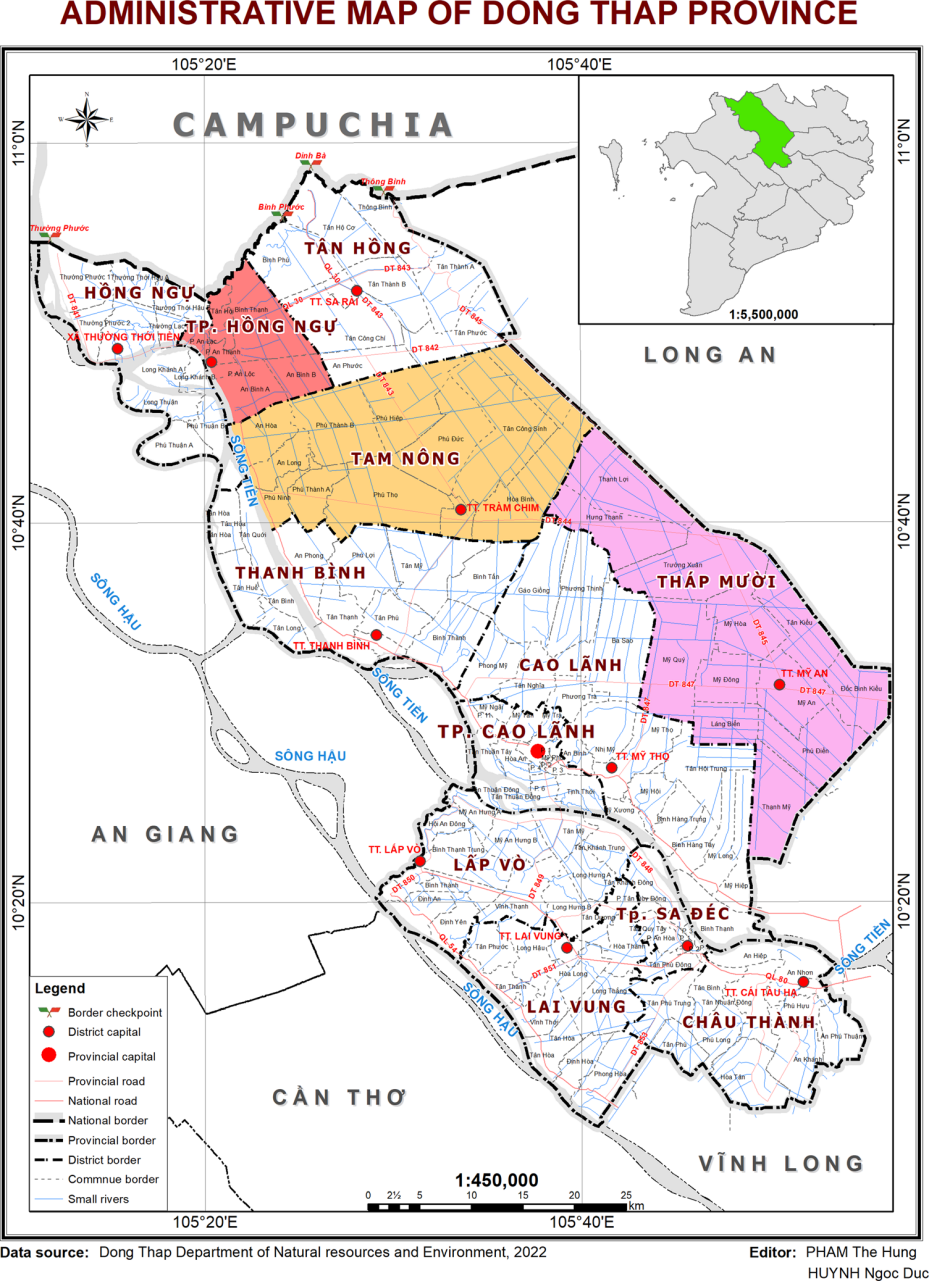
The study was conducted in the Tam Nong, Thap Muoi and Hong Ngu districts in the Dong Thap province, which are located in the upper part of the Mekong Delta in Vietnam

The study was conducted in the Hong Ngu, Tam Nong, and Thap Muoi districts in the Dong Thap province, which is located in the upper part of the Mekong Delta (Fig. 1). The province has fertile soil suitable for rice production, and is one of the provinces with the largest expansion of triple rice production in the Mekong Delta during the past two decades, which has been facilitated by protecting rice fields from seasonal flooding with high dikes (Tran et al. 2018). Approximately one third of the total cultivated area has three rice crops, but also two rice crops per year are applied by many farmers. All of the studied districts also have upland crops, orchards, vegetables and aquaculture (Tran et al. 2018).

### Survey of rice and rice-fish farming practices

Information about rice and rice-fish farming practices in the Dong Thap province of the Mekong Delta was collected during the spring of 2022, using Participatory Community Analysis (PCA), Key informant interviews with local officers and structured interviews, based on pre-tested questionnaires, with farmers from the Hong Ngu, Tam Nong, and Thap Muoi districts. The study was conducted in close consultation with local farmers as understanding farmer’s knowledge, attitudes and practices related to agriculture and the use of agriculture chemicals is crucial to assess health and environmental risks under different farming strategies (Galli et al. 2022). A mixed methodological approach was applied in this study, where quantitative and qualitative data collection were carried out to provide detailed information on farmer characterization and cropping pattern for three different farming systems;

A) Rice farming with two crops per year and low dikes (30 2RLd farmers), representing a “baseline”;
B) Rice farming with three crops per year and high dikes (30 3RHd farmers), representing “conventional intensification”;
C) Rice-fish farming with two rice crops and one fish crop per year (18 2RF), representing a “diversified intensification”.

When finished, the questionnaires were checked and translated by senior researchers from Vietnamese universities. Attributes of the interviewed farmers are found in Table 1. Data were checked for normal distribution, and when normally distributed differences between categories were investigated using one-way analysis of variance (ANOVA) with Turkey's HSD (honestly significant difference) used as the post-ANOVA test. When not normally distributed the nonparametric Mann–Whitney U test was used. SPSS for Mac (Ver 28.0,1; SPSS, Chicago, IL, USA) was used to analyze the data.

**Table 1:** Household composition, farm size and experience of rice and rice-fish farming among farmers in the Dong Thap provinces in 2022

		2RLd (30)	2RF (18)	3RHd (30)
Age of farm-owners (years)	Mean	53.7	51.0	53.0
	SD	10.7	10.7	9.3
Household size	Mean	3.8	4.4	4.6
	SD	1.2	1.1	1.5
No. of individuals involved in rice farming	Mean	1.5	2.0	1.8
	SD	0.7	0.9	0.8
Educational level (years)	Mean	7.3^a^	10.4^b^	9.3^ab^
	SD	3.4	4.0	2.1
Total farm area (ha)	Mean	2.4^a^	4.3^b^	2.6^a^
	SD	1.2	3.2	1.6
Experience in rice farming (years)	Mean	27.3	24.2	28.7
	SD	9.1	12.3	10.0
Experience in rice-fish farming (years)	Mean		2.4	
	SD		0.9	

Means that do not share the same superscript letter are significantly different (P < 0.05)

## Results

### Farming strategies and yields

There were no statistically significant differences in the rice yields per crop between the farmer categories, and farmers with three crops per year had statistically significant higher annual rice yields than farmers with two crops, because of the third crop (Table 2). Still, the majority of the farmers (70%) thought it was better to have two crops than three crops per year (Table S1). The main argument was that it allowed alluvium to enter the rice fields, which kept the soil fertile. Many of the 2RLd farmers (58%) said that this made the production more efficient by decreasing costs, increasing the productivity and thus the profit. A few 2RF farmers also experienced less pests. The only argument supporting three crops was that it provided a higher and more regular income (Table S1). However according to Table 3, there were no significant differences in the profits from the rice crop between 2RF farmers (59.6 mill. VND ha^−1^ year^−1^) and 3RHd farmers (58.7 mill. VND ha^−1^ year^−1^), and when adding the profit from fish, 2RF farmers had significant higher profit than 3RHd farmers (Table 4). The majority of the 2RF farmers (61%) had started with rice-fish farming because they thought this would increase their income through a more efficient production. 89% of these farmers had experienced a 20% increased income and 67% felt that fish had helped to increase the rice yields. 2RF farmers had the highest benefit cost ratio of the farming categories because of comparatively low cost and high yields, while 3RHd farmers had the lowest benefit cost ratio of the farming categories, because they used significant higher amount of rice seeds and agrochemicals per year than the other farmers, which resulted in statistically significant higher production costs and thus lower profit (Table 4).

**Table 2:** Annual use of fish and rice seeds, agrochemicals and yields of rice and fish (kg ha^−1^ year^−1^) in rice and rice-fish farming in the Dong Thap provinces in 2022

		2RLd (30)	2RF (18)	3RHd (30)
Rice Seed	Mean	301^a^	238^b^	342^c^
	SD	41	72	72
Rice Yield	Mean	13 882^a^	14 700^a^	20 206^b^
	SD	1502	1415	1648
Fish Stocking Density (fish m^−2^)	Mean		0.7	
	SD		0.5	
Yield of Farmed Fish	Mean		1219	
	SD		950	
UREA	Mean	200^a^	183^a^	534^b^
	SD	125	150	199
NPK	Mean	220^a^	247^ab^	111^b^
	SD	219	334	205
DAP	Mean	155^a^	143^a^	316^b^
	SD	109	100	151
K	Mean	87^a^	63^a^	194^b^
	SD	79	71	114
Total Fertilizer	Mean	664^a^	638^a^	1156^b^
	SD	237	200	247
Herbicides (a. i.*)	Mean	0.88	0.58	1.03
	SD	1.44	0.69	0.86
Fungicides (a.i)	Mean	0.99^a^	1.25^a^	2.49^b^
	SD	0.92	1.09	2.20
Insecticides (a. i.)	Mean	0.17^ab^	0.14^a^	0.43^b^
	SD	0.29	0.24	0.67
Molluscicides (a.i)	Mean	0.67	0.17	1.07
	SD	1.02	0.37	3.30
Total Pesticides (a. i.)	Mean	2.71^a^	2.14^a^	5.03^b^
	SD	2.09	1.05	4.30

Means that do not share the same superscript letter are significantly different (P < 0.05)

^*^Active ingredient

**Table 3:** Use of pesticides (% farmers) and number of different pesticides used in rice and rice-fish farming in the Dong Thap provinces in 2022

	2RLd	2RF	3RHd
Fungicides (%)	87	88	97
Herbicides (%)	47	59	73
Insecticides (%)	73	35	80
Mulluscicidies (%)	60	35	47
Rodenticides (%)	7	0	3
Increased use of pesticides (%)	17	6	17
Decreased use of pesticides (%)	33	83	60
Number of different pesticides used per farmer	4.1	3.6	5.0

**Table 4:** Costs and income (thousand VND ha^−1^ year^−1^) in rice and rice-fish farming in the Dong Thap provinces in 2022

			2RLd (30)	2RF (18)	3RHd (30)
Costs	Seed	Mean	4168^a^	3289^b^	4988^c^
		SD	537	853	915
	Fertilizer	Mean	8002^a^	7227^a^	15 149^b^
		SD	1813	1847	3826
	Pesticide	Mean	9390^a^	4340^b^	14 108^c^
		SD	5112	1384	3493
	Labor	Mean	8226^a^	9693^b^	12 430^c^
		SD	946	1170	4486
	Soil preparation		3502^a^	3650^a^	4114
		SD	962	787	544
	Water pumping		3383^a^	3035^a^	3570^b^
		SD	617	1028	121
	Harvest & transport		4132^a^	3976^a^	5968^b^
		SD	892	685	701
	Fish cost	Mean		22 597	
		SD		20 591	
	Total cost	Mean	40 803^a^	57 641^b^	60 328^b^
		SD	5451	21 216	7207
Income	Rice	Mean	87 296^a^	94 849^b^	119 067^b^
		SD	11 777	10 703	10 091
	Farmed fish	Mean		49 156	
		SD		28 513	
	Wild fish	Mean		7818	
		SD		10 541	
Total income		Mean	87 296^a^	151 823^c^	119 067^b^
		SD	11 777	30 548	10 091
Net income		Mean	46 493^a^	94 182^c^	58 739^b^
		SD	11 914	18 555	11 862
Benefit cost ratio		Mean	2.14^a^	2.63^b^	1.97^a^
		SD	0.37	0.65	0.30

Means that do not share the same superscript letter are significantly different (P < 0.05)

The majority of farmers with two rice crops felt that the rice yields had stayed stable or increased (74%), while 73% of the farmers with three crops felt the yields had decreased during the last five years (Table S1). The main reasons for this decrease were the lack of alluvium and decreased soil quality (55%) and bad unpredictable weather (45%). These were also the main reasons why 87% of the 3RHd farmers felt that the yields not would increase in the coming five years. Around 50% of the farmers with two crops felt that the yield would not increase, primarily because of unpredictable weather and reduced floods (Table S1). Reduced floods were also seen as the main cause (85%) of reduced fish yields among 2RF farmers and the main (78%) constraints for high fish yields in the future.

High production costs, especially for fertilizers were seen as the main constraint for rice farming in the future, and especially among 3RHd farmers (70%), while this was less commonly reported by 2RF farmers (28%). This is consistent with the fact that 2RF farmers were found to have the lowest production costs for rice, while 3RHd farmers had the highest production costs (Table 4). Also unpredictable weather/climate change and pests were seen as constraints by many farmers. A few 3RHd farmers also mentioned unfertile soils (Table S1).

The main environmental problems of rice farming perceived by the farmers were that it polluted the environment and decreased the soil quality. Lower use of agriculture chemicals and less intensive farming methods were proposed to be ways to solve these problems (Table S1).

Some 65% of all farmers felt that future farming strategies should aim for higher quality of rice rather than higher quantities, where several farmers with three crops suggested that this could be achieved by reducing the use of fertilizers and pesticides or by having two crops. Some also wanted to start with organic farming. The farmers got some 5% higher price for their rice if they used less pesticides. This was more commonly reported by farmers with two crops than by farmers with three crops.

### Pest management strategies

Pesticides were the most common way to control pests, especially among rice farmers, while 2RF farmers more frequently used other methods, aligned with IPM strategies (Table S2). Insects were the most problematic pests followed by bacteria and fungi. 2RF farmers used lower amount, and somewhat less toxic pesticides than rice farmers, and especially compared to 3RHd farmers (Table 2).

Farmers had learned how to use pesticides in many different ways and learning by doing (own experience) was most common among rice farmers, while advice from extension officers and other farmers were more common among 2RF farmers, which may have contributed to a more restricted use of pesticides among these farmers (Tables 2, S2, 4).

The majority of the 2RF farmers said that they had decreased their use of pesticides, and especially insecticides, during the last 3 years, and 2RF farmer used the lowest amounts of pesticides among the farmer groups (Tables 2 and S2). They also used pesticides less frequently and a lower number of different pesticides compared to the other farmers (Table 3). The changed use of pesticides among the other groups of farmers varied, and overall there was a comparatively small decrease of pesticide use among the 2RLd farmers. 3RHd farmers used statistically significant higher amount of pesticide per year than the other farmers, because of the additional third crop (Table 2). They also used a higher number of different pesticides compared to the other farmers (Table 4).

Health effects were the most common problem with pesticides mentioned by the farmers (Table S2). Also environmental pollution, and especially water pollution, was seen as a critical problem. Other problems mentioned included decreased number of natural enemies to pests, pest resurgence, increased production costs and decreased rice productivity, which all are interlinked effects of high use of pesticides (Table S2).

Some 70% of the farmers used some form of protection when using pesticides, and the most common included face mask (54%), shirt (31%) and hat (25%). Still, the majority of the farmers experienced health problems after using pesticides (> 90%), where many felt itchy and hot. These problems were most often felt after using insecticides (Table S2).

Most farmers changed the water level in their rice field before using pesticides. They often increased the water levels to treat insects like brown planthopper, which probably also helped to decrease the impact of pesticides on aquatic animals, while they decreased the levels to treat bacterial and fungi diseases. Thus, regulation of water seemed to be an important tool for improved pest control, which however seemed  to be a more limited option for 3RHd farmers, who did this less frequently (Table S2).

Almost half of the farmers decided to use pesticides based on field surveys and only a minority of the farmers relied on scheduled sprays. Many farmers also selected pesticides that only killed the target pest (Table S2). The majority of the 2RF farmers (95%) said that pesticides impacted on the fish yield and about half of the farmers thought that pesticides could have a negative effect on the rice yields, where the majority stated that excessive spraying was bad for the rice. Some also said that this led to resistant pests and killed natural enemies to rice pests, which could increase the pest problems in the field (Table S2).

Most farmers applied integrated pest management (IPM), and had learned about this from primarily extension workers, but also from media and other farmers. The most common example of IPM practices was a reduction in the use of pesticides, followed by a reduction in fertilizers and rice seeds (Table S2). The main reasons for applying IPM were to decrease production costs and to increase the productivity. Some farmers also felt this was an efficient way to control pests and limit the pesticide use. Most farmers felt that IPM had increased their income and especially 2RF farmers who felt that it had increased the income with 26%, while the income increase among 3RHd farmers was only 9% (Table S2).

### High dikes versus low dikes

The majority (76%) of farmers with two rice crops, and especially 2RF farmers, preferred low dikes, with the main argument that it allowed nutrient-rich sediments to enter the rice fields, so they could save costs for fertilizers, while still having a high productivity. Fewer pests were also seen as an advantage and problems with especially rats were more commonly reported in rice fields with high dikes (Table S3).

Still, 3RHd farmers seemed to be quite satisfied with high dikes, primarily because the dikes provided protection against floods, but also because of the possibility to have three crops per year, and thus generating a higher income (Table S3). Still around half of the farmers felt that high dikes could increase the risks for floods downstream, because when the river water cannot enter into the rice field it will flood other low-lying areas. The possibility of having orchards and that the dikes improved the road systems, were seen as additional advantages. The main disadvantages included high amounts of rats, low input of nutrient-rich alluvium, and less fish (Table S3).

Almost all farmers agreed or strongly agreed that high dikes decreased the soil fertility in the rice fields (Table S3). Approximately 70% of the farmers also agreed that high dikes could reduce the rice yields after some years. The majority of the 3RHd farmers (63%) had increased their use of ferilisers with 20%, and 30% of the farmers experienced 30% increased production costs, primarily for fertilizers (Table S3). Still half of the 3RHd farmers said that their income had increased because they now had one more crop. However, half of these farmers reported that their rice yield had decreased with some 14%. The main reason for this was less nutrient-rich alluvium and more pests (Table S3).

More than half of the farmers also said that high dikes had decreased the amounts of aquatic organisms very much and the majority felt that they had had an impact on biodiversity and fish yields (Table S3). This was partly seen as a consequence from decreased flow of water and connectivity. 60% of the 3RHd farmers said that high dikes had affected the water quality in the rice field through less nutrient-rich alluvium and because a high use of agrochemicals (Tables 3, S3).

## Discussion

The Mekong Delta has been of critical importance for rice production for decades and will most likely continue to be so (Dang et al. 2021; Lan and Nguyen. 2021). Seasonal flooding has provided the Delta with nutrient-rich sediments and water creating optimal conditions for agricultural and aquaculture productivity (Dung et al. 2018). Kondolf et al. (2018) estimated that the Mekong Delta produces around 2.4% of the global rice harvest and 0.5% of the total global calorie supply*.* The natural high productivity has been further increased by high use of agrochemicals and the construction of high dikes to protect crops. This has helped to increase rice yields but also disrupted mechanisms that sustain the natural productivity of the Delta, and during the last decade, the need to develop food production systems that not only produce high yields but also helps to meet future needs of different stakeholders in the Delta, has increasingly been discussed (Vietnamese Government 2017, 2020; Nguyen et al. 2018; Tran et al. 2018; Nguyen et al. 2019; Tran et al. 2019; Lan and Nguyen 2021; Hui et al. 2022).

The Mekong Delta is currently facing major challenges in terms of climate change and upstream dams and current food production systems should be designed so they contribute to an increased resilience and a sustained production of food (Vietnamese Government 2017, 2020; Spangenberg 2019; Hui et al. 2022). The Delta is at cross roads where future farming strategies either could focus on an intensified production of rice through increased input of external resources, such as more agriculture chemicals, in an increasingly human controlled environment, or build on a diversified production of rice and other crops, with the aim to increase the efficiency use and recycling of resources rather than a throughput of external resources. Such a transition of food production systems should aim to meet the diverse needs of people and build on an effective use of ecosystem services in ways that are regenerative and minimizing negative impacts. This requires optimal management of nature’s ecological functions and biodiversity to improve agricultural system performance, efficiency and farmers’ livelihoods.

### Farming strategies and yields

Based on our results we argue that a transformational change of current agriculture practices in the Mekong Delta is possible and that it would make economic, ecological, and social sense in the long-term. As shown in this study and previous studies, options for alternative farming strategies, that would be more sustainable than three crops of rice with high dikes are available in the Mekong Delta (Tran et al. 2018; Lan and Nguyen 2021). Many farmers are willing and flexible to adapt to such methods if appropriate support is provided (cf. Spangenberg 2019). However, the adapting capacity of the Delta is decreasing (Smajgl 2015). 3RHd farmers, for example, have limited their options to develop profitable crop diversification, which relies on natural productivity mechanisms, partly because of a reduced connectivity between the rice field ecosystem and the environment (cf. Berg et al. 2016; Tran et al. 2018). For these farmers fertilizers and pesticides have become necessary fossil-based substitutes to compensate for the losses of natural ferilization and pest control processes, at increasing costs. Thus, our findings support previous research that shows that triple rice production within high-dike systems may not only be ineffective in increasing farmers' profits and livelihoods in the long-term, but also worsen farmers health and environmental degradation, due to high use agrochemicals and disrupted connectivity within and between rice field ecosystems (Chapman and Darby 2016; Tran et al. 2018, 2019; Nguyen et al. 2018).

Integrated farming systems, such as rice-fish farming, on the other hand builds on an increased connectivity within the rice field ecosystem, where the fish have several positive effects on rice by feeding on rice pests, providing nutrients and improving the soil quality (Xie et al. 2011; Zheng et al. 2016). These positive feed-backs between rice and fish, confirmed by many 2RF farmers, may explain the comparatively high rice yield of 8.2 tons per hectare for the first rice crop among the 2RF farmers. This was higher than for the other farmers and the annual yield of rice in rice-fish farming was surprisingly high considering the comparatively low input of rice seeds and agrochemicals. This high productivity, based on an efficient internal recycling of nutrients, should be compared with triple rice cropping in high dikes, where the main concerns, repeatedly raised by the 3RHd farmers, were the lack of nutrient-rich alluvium and fertile soil.

The main argument for farmers to have three crops was that it provided them with more stable and higher income. However, our financial analysis shows that farming with two crops of rice and one crop of fish provides a higher net income. In fact, the benefit cost ratio is the lowest for 3RHd farmers. This is primarily because they have significant higher (50–70%) production costs than the other farmer groups, which is similar to the findings by Tran et al. (2018) and Chapman and Darby (2016), who found that farmers profit with triple rice crops diminished over time. On a regional scale Tran et al. (2019) showed that the expected net agriculture profit from a scenario up to 2030 with two crops and low dikes would be much higher than a scenario with three crops and high dikes, and concludes that intensive rice production under high dikes is financially unfeasible and unsustainable in the long run. Even the advantage of flood protection in upstream areas, mentioned by some farmers, must be questioned as the majority of farmers said that high dikes can lead to increased flood risks in downstream areas (Tran et al. 2018; Hui et al. 2022).

Although 3RHd farmers had a higher annual yield of rice, this should be balanced against having another crop of fish. A diversified production helps to spread the risks of crop failures and could in the long-term provide a more stable income than triple rice cropping, as failures in one crop can be compensated by the other crop (Pretty and Bharucha 2014; Alam et al. 2022). As indicated in the interviews with farmers, many were increasingly experiencing problems with pest outbreaks and unpredictable weather, partly due to climate change, and there is a need to make farming systems more resilient through enhanced diversity in land use at the farm level, which could help to secure more stable yields in the future (Spangenberg 2019).

The majority (73%) of the farmers with three rice crops said that the rice yield had decreased during the last five years, which was much more than for farmers with two rice crops, where the yield primarily had been stable or increased. 3RHd farmers saw unstable prices for rice and agrochemicals as major challenges for rice farming, which also were perceived as the main risks among rice farmers in An Giang (Dang and Pham 2022), and the low marginal profit of rice make 3RHd farmers more vulnerable to price fluctuations than rice farmers with two crops (Tran et al. 2018). Thus, considering both ecological (e.g. pest outbreaks) and financial factors (e.g. market fluctuations), three rice crops are unlikely to provide a more stable income than two rice crops (Chapman and Darby 2016), which is in accordance with the findings of Tran et al. (2018), who concluded that triple rice production over time would result in both environmental and economic losses in the Mekong Delta.

Crop diversification, on the other hand, helps farmers offset the market risks associated with rice monocropping and helps farmer to diversify their livelihoods and income (Pretty and Bharucha 2014; Alam et al. 2022). The majority of the 2RF farmers felt that rice-fish farming had increased their profit, both because of decreased costs but also because of increased yields of rice and fish. Farmers with two crops, and especially those integrating fish and applying IPM, are also in a better position to benefit from the increased demand and prices for environmentally certified rice, because of their lower use of agrichemicals (cf. Khai and Jabe 2015; Lan and Nguyen 2021).

### Pest management strategies

3RHd farmers used significant higher amounts of pesticides than the other farmer groups, which is similar to the finding by Tran et al. (2018). Pesticides were more commonly used among these farmers, who also used a higher number of different pesticides, than farmers with two rice crops, and especially compared to 2RF farmers. This higher dependence on pesticides compared to the other farmers, could be due to re-enforcing loops, where an increased use of pesticides had decreased the number of natural enemies, which in turn requires more pesticides. This is a common problem in intensive rice farming in the Mekong Delta and combined with an overuse of nitrogen fertilizers and three crops of rice, pests can flourish in an environment with a constant supply of food and free of enemies (Heong et al. 2015; Yuen et al. 2021). At this stage a high use of insecticides is often necessary for the farmers to control pests, which can explain the comparatively high and common use of insecticides among 3RHd farmers (Tables 2, 4). The high use of pesticides also increases the risk for pesticides resistant pests, which in turn requires new pesticides. Thus, a high use of pesticides and three crops of rice easily lead to a decreased connectivity between rice pests and their natural enemies and create a high dependence on pesticides, which in the long run may be more expensive and tentatively less efficient than natural pest control mechanisms (Heong et al. 2015).

2RF farmers on the other hand relied to a larger extent on biological control methods and IPM, where fish helped to control rice pests when using them for feed (Xie et al. 2011), which could explain why these farmers used less pesticides than the other farmers (Table 2). These farmers were also more concerned than the other farmers that pesticides killed natural enemies and could have negative impacts on the fish. This contributes to a reduced use of pesticides, as the potential impact on fish often is considered as an additional cost of pest control, shifting the economic threshold for applying pesticides to a level which is higher than for farmers who aims to only maximize the yield of rice (Berg 2001).

The low use of pesticides also resulted in comparatively low costs for pesticides among 2RF farmers, whose profit was further strengthened by the fact that many consumers in the Mekong Delta are willing to pay a higher price for rice produced with less pesticides (Khai and Jabe 2015). Altogether these factors seem to have increased 2RF farmers awareness of the impacts of pesticides on the environment, creating both ecological and financial incentives to minimize the use pesticides (Berg 2002). This shift in attitude could explain why more 2RF farmers applied IPM than the other farmers. They also seemed to be more successful in IPM and stated that this had increased their income with some 26%, and that this was an effective way to control pests with less pesticides, which helped to reduce costs. As argued above and in earlier studies, IPM and rice-fish farming are, thus, complementary activities, which increase each others efficiencies, outcomes and implementation rates (Berg 2002; Berg et al. 2012).

These biodiversity-based farming strategies provide concrete and important guidance on how future agriculture activities can build on enhanced use of ecosystem services, biodiversity and connectivity for a sustainable and healthy production of food, which is less dependent on synthetic agrochemicals and more resilient to future environmental changes (cf. Spangenberg 2019; Lan and Nguyen 2021). Such ecological approaches to food production tend to be knowledge-intensive processes, requiring optimal management of nature’s ecological functions and biodiversity to improve agricultural system performance, efficiency and farmers’ livelihoods. Similar to earlier studies, 2RF farmers had slightly higher education levels than the other farmers, which could explain why these farmers had adopted rice-fish farming and IPM (Berg and Tam 2012, 2018). These farmers also relied more on extension officers for advice on how to use pesticides. Thus, support to capacity and knowledge building is crucial, not only to improve technical knowhow, but also to facilitate a transformational mind-shift of farmers as well as policy makers, which can be enhanced by a strong social connectivity (Garbach et al. 2014; Pretty and Bharucha 2014).

A more restricted use of pesticides also helps to decrease health effects, which were seen as the most critical problem with pesticides. More than 90% of the farmers experienced health effects from using pesticides, which is similar to findings from earlier surveys (Berg 2001; Berg and Tam 2012, 2018). Although many farmers used some protection these protections are often not enough and farmers will most likely continue to be affected by pesticides in the future, as many are reluctant to wear full protection because they feel uncomfortable (Galli et al. 2022). Thus, an important way to reduce health risks from pesticides would be to adopt farming strategies that encourage a decreased use of pesticides and increases farmers awareness of their potential negative effects on producers and consumers health and the environment. (cf. Galli et al. 2022).

### High dikes versus low dikes

Many studies have shown that the expansion of high-dike agricultural systems have led to reduced inflow of nutrient-rich sediments and water, which was confirmed in our study (Nguyen et al. 2018; Tran et al. 2018, 2019; Hui et al. 2022). For 3RHd farmers high production costs, primarily for fertilizers, were seen as major constraints for future rice farming. Almost 90% of all farmers said that high dikes had decreased the soil fertility and 50% thought that the decrease was very strong, because the high dikes blocked nutrient-rich river water and sediments to enter the rice fields. More than 50% of the 3RHd farmers experienced that their rice yields had decreased, primarily because of less alluvium and more pests, and the majority thought that the yields not would increase in the future because of unfertile soil. 60% of these farmers had increased their use of fertilizers and many experienced increased production costs*.* Only 50% of these farmers said that the third crop had increased their income, while 20% had experienced a decreased income. These results support earlier studies, which found that the use of agrochemicals and the negative effects from high dikes increase with time and that the long-term financial sustainability of three crops with high dikes must be questioned (Nguyen et al. 2018; Tran et al. 2018, 2019).

The majority of the farmers also felt that high dikes had decreased the amounts of aquatic organisms, especially fish and that this had decreased the biodiversity because a loss of connectivity and habitats (cf. Dang et al. 2021). Nguyen et al. (2018) found significant decreases in the numbers of native fish and vegetables in areas enclosed by high dykes in An Giang. This has serious implication for food security and peoples’ livelihoods, as wild foods are critical sources of protein, energy and micronutrients, especially for those most vulnerable. (Pretty and Bharucha 2014; Chapman and Darby 2016; Nguyen et al. 2019). As reported by Nguyen et al. (2019), high dikes in the upper parts of the Mekong Delta have severely reduced the habitats available to freshwater fish and other aquatic organisms, diminishing the catches of wild fish and aquatic animals that are crucial food sources for the poor. Thus, dike heightening risks to penalize poor and landless people, as it disrupts their access to flood-based livelihoods. The limited exchange of water increases the pressure on aquatic biodiversity even further and 60% of the 3RHd farmers felt that high dikes had affected the water quality, primarily through increased pollution with pesticides and fertilizers, but also through less input of nutrient-rich alluvium (cf. Nguyen et al. 2018).

In comparison, rice-fish farming encourages and builds on an increased aquatic biodiversity, where both farmed and wild fish and other aquatic organisms, are seen as important components of integrated systems that contribute to a high yield through an enhanced reliance on ecosystem services. It manages agricultural areas as multi-functional dynamic systems where high connectivity and diversity are key assets to make these systems operate well (cf. Pretty and Bharucha 2014). Annual inflows of nutrient-rich flood water were, for example, seen as a main benefit of having low dikes and crucial for the long-term yield of rice and fish, helping farmers to increase their profits through decreased production costs and increased income*.* Fluctuating water levels were used to optimize farming practices and yields and many farmers changed, for example, the water levels in the field before applying pesticides to both protect the fish and to increase the impact on target pests. Thus, a regular exchange of water combined with efficient recycling of nutrients and organic matter help to decrease the use of agrochemicals, improve the water quality and stabilize interlinked food-webs that contributes to pest control and food production.

In conclusion, this study shows that rice farming with three rice crops and rice-fish farming with two rice crops are quite different systems, although both aim to produce rice and increase the production of food in the Mekong Delta. Systems with three crops and high dikes are designed primarily for a high production of rice in a highly controlled environment, supported by high inputs of fertilizers and pesticides, and where natural mechanisms increasingly are substituted by external fossil fuel supported inputs. Although this may increase the rice yield in the short-term it comes with increasing financial, environmental and social (health) costs*.*

Intensive rice monocropping also creates systems which are increasingly vulnerable to external disturbances, such as pests, climate change and unstable markets for rice and agrochemicals, which all were seen as major constraints to rice farming and of major concern to the farmers. One reason for this is a decreased diversity and connectivity, which often is followed by a decreased systems resilience. Considering that the Delta is already under high stress from multiple external factors*,* strengthened resilience of agriculture and natural systems should be of high priority, since people depend on healthy, functioning ecosystems for a long-term agriculture production (Spangenberg 2019). Thus, a fundamental element for future farming strategies in the Mekong Delta should be to bring diversity and complexity back into the agricultural landscape, where a challenge will be to create diverse farming systems that are productive, resilient and enablers of ‘intensification without simplification’(cf. Pretty and Bharucha 2014).

These strategies are in line with government policies during the last decade to promote more sustainable agriculture practices (Vietnamese Government 2017, 2020). As found in this study, small holder farmers in the Mekong Delta are often open and flexible to adopt new farming strategies that would improve the quality of rice, the environment and their health, even if it would imply some decrease in the yield (Berg et al. 2016; Lan and Nguyen 2021; Galli et al. 2022). However, the long-term policy trend in the Delta has been to encourage highly productive monocultures, and it has for many decades been suspected that the promotion of biodiversity while reducing reliance on agrochemical inputs, would be penalizing yields on a regional scale (Spangenberg 2019). Thus, further efforts are needed to support a transformational change of agriculture practices in the Mekong Delta, building on both existing integrated productions system but also supporting experimentation and pilot projects at farm levels (cf. Tran et al. 2019).

With appropriate technical and policy support, this could pave the way for large scale agriculture transformations, but time is getting scarce as external disturbances, such as climate change and upstream hydropower dams, and internal factors, such as agriculture and aquaculture intensification are quickly limiting future opportunities.

## Conclusion

This study shows that integrated rice-fish farming could provide sustainable alternatives to more intensive rice monocropping strategies for increased and diversified food production in the Dong Thap province in the Mekong Delta. Financially farms with two crops of rice and one crop of fish were shown to provide a much higher annual net income than farms with three crops of rice and high dikes. This was primarily due to lower production costs for agrochemicals and high yields of rice and fish. Ecologically, low dikes and low use of pesticides and fertilizers create less impacts on the environment, and also allows for a higher biodiversity, stimulating a more efficient circulation of nutrients and natural control mechanisms of rice pests, which are further strengthened by the farmed fish. Socially less pesticides, a diversified production of crops and increased income improve the farmers health and wellbeing. All these factors are interlinked and tend to enhance each other through positive feedbacks. A high connectivity both within and between farming systems are crucial for a high and stable production of healthy food and for building farming systems with a high resilience to future environmental changes. More diverse and less intensive agriculture systems, such as integrated rice-fish farming, could contribute to a sustainable intensification of food production in the Mekong Delta.

## Supplementary Information

Below is the link to the electronic supplementary material.

Supplementary file1 (PDF 39 kb)
