# Supplementary material for: An ecological economic comparison between integrated rice-fish farming and rice monocultures with low and high dikes in the Mekong Delta, Vietnam
Source: Ambio. 2023 May 15;52(9):1462–74. doi: 10.1007/s13280-023-01864-x (PMC10406997; doi:10.1007/s13280-023-01864-x)
Supplement: Supplementary file 1 — Supplementary file1 (PDF 39 kb) [file 13280_2023_1864_MOESM1_ESM.pdf]

Ambio
Electronic Supplementary Information
This supplementary information has not been peer reviewed.

Title: An ecological economic comparison between integrated rice-fish farming and rice monocultures with low and high dikes in the Mekong Delta,
Vietnam

Authors: Håkan Berg, Thai Huynh Phuong Lan, Nguyen Thanh Tam, Duong Huyen Trang, Pham Huynh Thanh Van, Huynh Ngoc Duc, Chau Thi Da

Table S1. Answers (% of famers) related to farming strategies among rice and rice-fish farmers in the Dong Tap province in 2022*

	2RLd	2RF	3RHd
1. Do you think two or three crops of rice are best?			
Two crops, because;	80	94	40
More alluvium & fertile soils	46	65	75
Higher productivity & profit	58	18	25
Less pests	0	29	0
Three crops, because;	13	6	50
Higher income	100	100	100
2. Has the rice yields changed during the last 5 years?			
Increase	33	22	10
Decrease	23	28	73
No change	43	50	17
3. Do you think the rice yields will increase in 5 years?			
Yes	50	50	13
No	43	50	87
4. What are the major constraints to rice farming?			
High production costs (fertilizers)	50	28	70
Unpredictable wheather	33	39	33
Pests	10	28	27
Unfertile soils	0	0	10
5. What is the most critical impact of rice faming on the environment?			
Environmental pollution	63	78	67
Less fertile soils	27	33	27
Less natural enemies & more pests	7	11	10
No effect	3	0	20
6 How can this impact be decreased?			
Reduced agrochemical use	47	39	50
Less intensive farming	7	28	17
7. Should future strategies aim for increased rice quality or quantity?			
Quality	60	61	73
Quantity	27	22	23

	2RF
8. Has rice-fish farming changed your income?	
Yes	89
No	11
If yes, how much (%)?	20
9. Have fish catches in rice field changed during the last three years?	
Increase	18
Decrease	59
No change	18
10. If decreased, which is the main reason?	
Change in flooding	86
Overfishing	14
Pollution/pesticides	14
11. Do the fish influence on the rice yield?	
Increase	67
No change	28
12. Are your fish farming practices affected negatively by pesticides?	
Yes	94
No	6
13. What do you see as the major constraint to rice-fish farming in the future?	
No flood water (makes fish grow slowly)	78
Other	22

*As farmers often provided more than on answer (and sometimes no answer) the percentage do not always add up to 100%

Table S2. Answers (% of famers) related to pesticides and pest management strategies among rice and rice-fish farmers in the Dong Tap province in 2022*

	2RLd	2RF	3RHd
1 Which methods do you use to control pests?			
Pesticides	100	89	100
Biological control	17	28	7
Pest resistant rice varieties	0	11	3
Traps	10	6	3
2. Which are the most problematic pests?			
Insects	77	72	70
Diseases/fungi	27	67	60
Bacteria	60	44	47
Snail	60	50	37
Weeds	33	33	27
Others (including rats)	3	11	33
3. Where did you learn how to use pesticides?			
Own experience	67	50	63
Extension officer	63	67	30
Other farmer	53	67	37
Media	47	56	30
Pesticide agent	33	33	30
4. Any problems related to pesticides?			
Human health effects	90	94	87
Environmental pollution	83	89	80
Kill natural enemies	63	78	50
Increase production costs	53	67	47
Pest resurgence	23	44	43
Decrease rice productivity	23	22	0
5. Have pesticides been a problem for your health?			
Yes	93	94	93
No	3	6	7
6. If health problem. Which problems?			
Itchy/hot	39	41	57
Fatigue	29	29	11
Respoitory problems	29	12	21
Dizzy	4	0	25
Headace	4	0	14
From which pesticides?			
Insecticide	82	76	79
Herbicide	25	53	29
Fungicides	11	18	39
Molluscicides	18	6	39

	2RLd	2RF	3RHd
8. Do you make any changes in the rice field before spraying?			
Yes (change water level)	83	72	53
No	13	28	27
9. How do you decide to spray pesticides?			
Field survey	93	89	87
Extension officer	23	33	33
Pesticide agent	27	28	13
Other farmers	30	17	17
Scheduled spray	20	17	3
Other	0	6	10
10. How do you select pesticides?			
Pesticide that only kill target pest	93	83	97
Pesticide agent	63	67	17
Extension officer	30	56	30
Personal experience	37	28	30
Other farmer	23	33	10
11. Do you think pesticides can have a negative effect on the rice yield?			
Yes	60	44	46
No	40	56	54
If yes, why?			
Excessive use is bad for rice	28	25	77
Kill natural enemies	17	38	8
More resistent pests	22	0	0
Decrease soil quality	11	0	0
12. Do you apply IPM?			
Yes	73	94	83
No	13	6	17
13 Example of IPM			
Reduce pesticides	100	65	76
Reduce fertilisers	91	40	60
Reduce seeds	77	41	60
14. Reason for applying IPM ?			
Increase income	18	0	0
Reduce cost	50	29	60
Increase productivity	36	12	36
Use less pesticides	0	18	12
15. Has IPM changed your income?			
Yes	77	94	96
No	23	0	4

7. Do you use any protection when spraying?			
Yes	63	67	87
No	27	28	13
What kind of protection?			
Face mask	79	67	92
Hat	32	42	15
Shirt	26	67	27
Glove	0	8	35

% increase	19	26	9
SD	6	13	7

*As farmers often provided more than on answer (and sometimes no answer) the percentage do not always add up to 100%

Table S3. Answers (% of famers) related to dikes among rice and rice-fish farmers in the Dong Tap province in 2022*

	2RLd	2RF	3RHd
1. Do you prefer high dike or low dike?			
High dike	33	6	87
Low dike	63	89	13
Arguments supporting low dikes:			
Low dikes provide alluvium	53	44	25
Higher productivity and lower costs	42	13	25
Less pests	16	6	0
Arguments supporting high dikes:			
Flood protection	40	0	38
Can have three rice crops	30	0	15
Higher income	0	0	27
2. What is the benefits of high dikes?			
Higher income/3 crops	63	50	27
Protection from floods	30	17	63
Possible to have orchards	0	11	13
Improve the road-system	0	0	13
3. What is the disadvantage of high dikes?			
More rats (pests)	53	22	7
No alluvium	40	56	40
Less fish	7	6	0
4. What effects can high dikes have on soil fertility?			
No effect	17	6	10
Some decrease	33	33	47
Very strong decrease	50	56	40
5. What are the effects of high dikes on aquatic organisms including wild fish?			
No effect	20	6	20
Some decrease.	23	28	20
Very strong decrease	57	56	53
6. Does high dikes have an impact on biodiversity?			

	2RLd	2RF	3RH d
7. Can high dikes increase floods downstream?			
Yes	47	50	50
No	23	33	43
If yes, why?			
Water flood lower areas	18	7	50
8. Can high dikes reduce the rice yields after some years?			
No	17	11	27
Agree	43	28	43
Strongly agree	33	44	27
9. Have high dikes changed your production costs?			
Increase			30
Decrease			13
No change			57
10. Have high dikes changed your income from rice?			
Increase			50
Decrease			20
11. Have high dikes changed your rice yields?			
Increase			3
Decrease			53
If decrease, why?			
Less alluvium, unfertile soils			56
More pests			13
12. Have high dike changed your use of fertilisers?			
Increase			63
Decrease			0
No change			37
If increase, why?			
Less alluvium, unfertile soil			47
13. Do high dikes affect the water quality in your ricefield/area?			

Yes	57	78	53	Yes	60
No	30	6	40	No	37
How?				*I yes, why?*	
Less aquatic organisms/fish	18	50	25	More polluted with fertilizers and pesticides	44
Loss in connectivity/habitats	6	0	31	Less alluvium	33

*As farmers often provided more than on answer (and sometimes no answer) the percentage do not always add up to 100%
